# Functional analysis of Cwc24 ZF-domain in 5′ splice site selection

**DOI:** 10.1093/nar/gkz733

**Published:** 2019-08-28

**Authors:** Nan-Ying Wu, Soo-Chen Cheng

**Affiliations:** Institute of Molecular Biology, Academia Sinica, Taipei, Taiwan 115, Republic of China

## Abstract

The essential splicing factor Cwc24 contains a zinc-finger (ZF) domain required for its function in splicing. Cwc24 binds over the 5′ splice site after the spliceosome is activated, and its binding prior to Prp2-mediated spliceosome remodeling is important for proper interactions of U5 and U6 with the 5′ splice site sequence and selection of the 5′ splice site. Here, we show that Cwc24 transiently interacts with the 5′ splice site in formation of the functional RNA catalytic core during spliceosome remodeling, and the ZF-motif is required for specific interaction of Cwc24 with the 5′ splice site. Deletion of the ZF domain or mutation of the conserved ZF residues greatly weakened the association of Cwc24 with the spliceosome, and lowered the affinity and specificity of its interaction with the 5′ splice site, resulting in atypical interactions of U5, U6 and Prp8 with the 5′ splice site, and aberrant cleavage at the 5′ splice site. Our results reveal a crucial role of the Cwc24 ZF-motif for defining 5′ splice site selection in the first splicing step.

## INTRODUCTION

The spliceosome catalyzes the removal of intervening sequences from precursor mRNA in a two-step process. It consists of five small nuclear RNAs (snRNAs), U1, U2, U4, U5 and U6, and a range of protein factors (1–3). The snRNAs play roles in recognizing short conserved sequence stretches in the 5′ splice site (5′SS) and branch site (BS) regions of the intron through RNA–RNA base pairings, which also form the basic framework of the spliceosome catalytic core. Although they do not directly participate in catalytic reactions (4), the protein factors help stabilize the RNA structure and mediate structural changes of the spliceosome along the splicing pathway. Eight DExD/H-box ATPases are required for the splicing reaction. They utilize the energy from ATP hydrolysis to drive structural changes of the spliceosome and facilitate splicing progression. Except for Brr2, all of these DExD/H-box ATPases interact only transiently with the spliceosome at specific steps of the splicing pathway (5–8). Prp8 is a core component of the spliceosome that interacts with the 5′ splice site, the 3′ splice site (3′SS) and the branch site of the pre-mRNA (9–17), as well as with several protein components on the spliceosome, thus playing a key role in mediating the splicing reaction (18).

**Figure 1.**
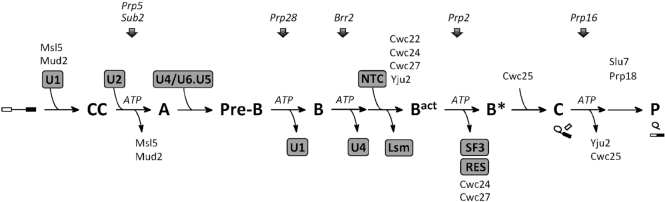
Schematic for the spliceosome assembly pathway showing splicing factors recruited to and released from the spliceosome at each stage. Cwc22, Cwc24, Cwc27 and Yju2 are recruited to the spliceosome immediately after or concomitant with binding of NTC. Slu7 and Prp18 are required for the second catalytic step, but can be recruited to the spliceosome at earlier steps. DExD/H-box proteins, Prp5, Sub2, Prp28, Brr2, Prp2 and Prp16, required for transition between specific steps are also shown.

The spliceosome is a highly dynamic structure that is assembled by sequential addition and removal of snRNAs and specific protein factors to the pre-mRNA. During spliceosome assembly, U1 first binds to the 5′ splice site and U2 binds to the branch site. Following binding of the U4/U6-U5 tri-snRNP, the spliceosome undergoes a major structural rearrangement to release U1 and U4 before forming new base pairs between U2 and U6, and between U6 and the 5′ splice site. The Prp19-associated complex (NTC for NineTeen Complex) is then added to the spliceosome, which is required to stabilize the interactions of U5 and U6 with the pre-mRNA during formation of the active spliceosome (19–21). Despite all essential base pairs of the RNA catalytic core having formed after the spliceosome is activated, the branch helix is still about 50 Å away from the 5′ splice site, sequestered by the U2 components SF3a/b (22,23). The DExD/H-box ATPase Prp2 and its cofactor Spp2 are required to remove SF3a/b from the spliceosome to allow the interaction of the branchpoint and the 5′ splice site so that the catalytic reaction can take place (24,25). By interacting with the C-terminal domain of Brr2, Prp2 is recruited to the spliceosome and is then translocated onto the pre-mRNA in the region ∼25 bases downstream of the branchpoint (26). By means of ATP hydrolysis, Prp2 moves along the pre-mRNA in the 3′ to 5′ direction toward the branch site to displace SF3a/b from the spliceosome (26). Accompanying SF3a/b dissociation, Cwc24 and Cwc27 are also released from the spliceosome (27,28). Cwc25 is then recruited to the spliceosome to promote the branching reaction, but is immediately released together with Yju2 after branch formation. Slu7 and Prp18 are then required to promote exon ligation. Schematic of the spliceosome assembly pathway is shown in Figure 1.

**Figure 2.**
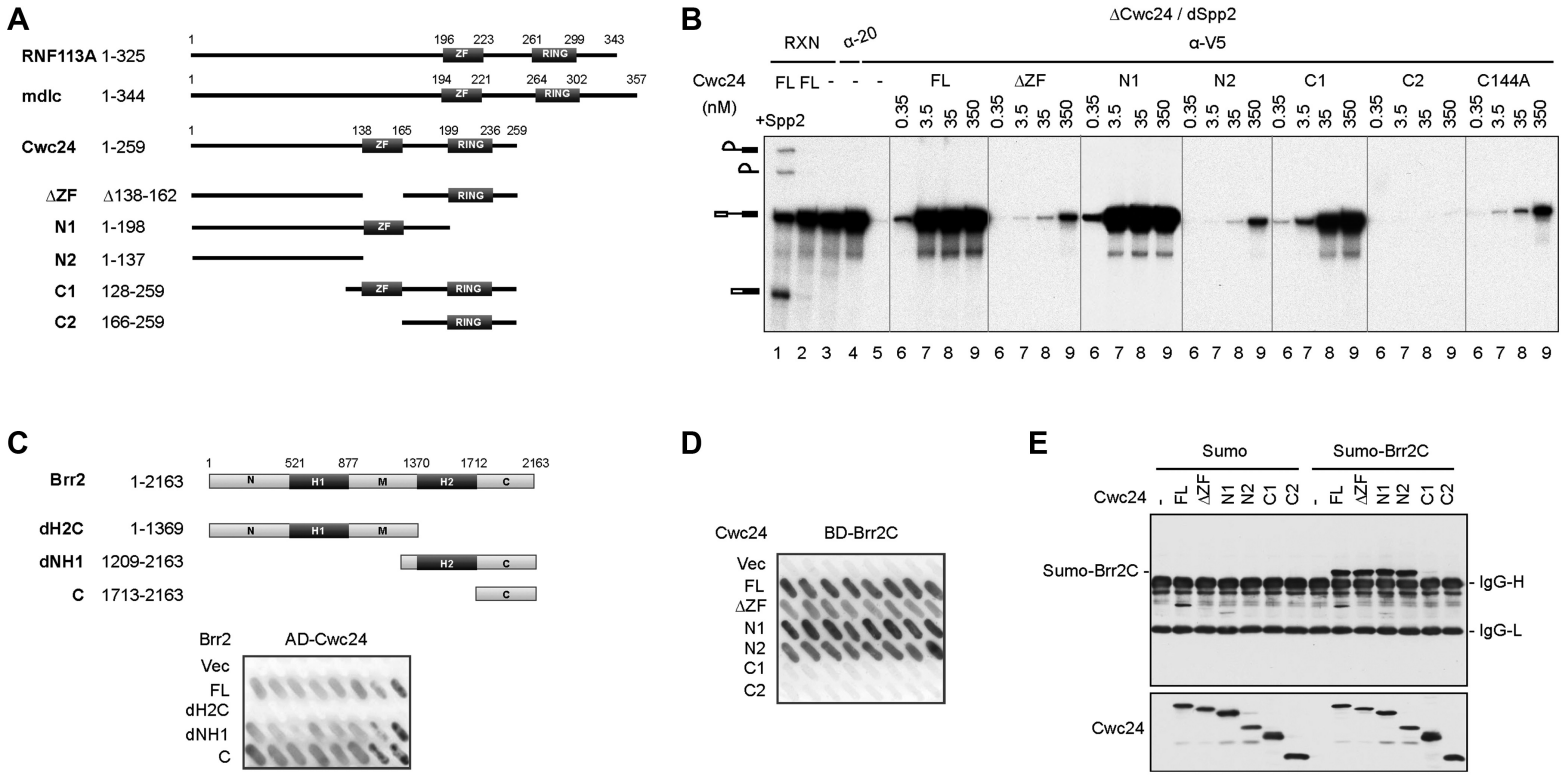
The ZF domain is required for the interaction of Cwc24 with the spliceosome, but not with Brr2. (**A**) Schematic representations of domain structures of Cwc24 and its human (RNF113A) and Drosophila (mdlc) orthologs, and Cwc24 deletion mutants. (**B**) Splicing reactions were carried out in Spp2-depleted ΔCwc24 extracts supplemented with recombinant V5-tagged Cwc24 proteins at final concentrations of 0.35, 3.5, 35 and 350 nM. The reaction mixtures were precipitated with anti-V5 antibody. The control reaction was precipitated with anti-Ntc20 antibody. ΔCwc24, extracts metabolically depleted of Cwc24; α-20, anti-Ntc20; FL, full-length. (**C**) Two-hybrid interactions of Cwc24 with segments of Brr2 fused to the DNA-binding domain. AD-Cwc24, Cwc24 fused to the activation domain; Vec, vector; FL, full-length. (**D**) Two-hybrid interactions of Brr2C with deletion mutants of Cwc24 fused to the activation domain. BD-Brr2C, Brr2C fused to the DNA-binding domain. Vec, vector; FL, full-length. (**E**) Interactions of Cwc24 deletion mutants with Brr2C were analyzed by immnuoprecipitation with anti-V5 antibody against bacterially expressed V5-tagged Cwc24 and His-SUMO-tagged Brr2C, followed by western blotting probed with anti-His (upper panel) and anti-V5 (lower panel) antibodies. IgG-H, IgG heavy chain; IgG-L, IgG light chain.

Cwc24 is an essential yeast protein that was previously isolated in a complex associated with Cef1/Ntc85 (29). It contains a zinc-finger (ZF) and a RING domain. Sequence alignment of Cwc24 orthologs reveals that the budding yeast Cwc24 represents a short form of the protein in comparison with those of other organisms from the fission yeast to human, containing only the core region (Figure 2A). The *Drosophila* ortholog of Cwc24, midlife crisis, is required to maintain neuronal differentiation (30), and mutations in its human ortholog, RNF113A, cause X-linked trichothiodystrophy (31). We have previously shown that Cwc24 is required not only for stable association of Prp2 with the spliceosome, but also for proper interactions of U5 and U6 with the 5′ splice site. Deletion analysis further revealed that the ZF domain is essential for cellular growth and for the *in vitro* splicing reaction, whereas the RING domain is dispensable (32).

Classical ZF domains comprise around 30 amino acid residues. They are extremely abundant in complex organisms and are typically involved in nucleic acid binding and/or protein–protein interactions (33). Cwc24 hosts a C3H-type ZF motif, in which three cysteine residues and one histidine residue are essential for proper coordination of the zinc ion to establish a rigid conformation. Point mutations in the invariable cysteine and histidine residues within the ZF result in significant changes to the secondary structure of the polypeptide, as observed by circular dichroism spectroscopy (34), indicating that amino acid substitutions within the C3H-type ZFs disrupt its structural integrity. We have previously shown that a single point mutation in the ZF domain, changing cysteine 144 to alanine (C144A), abolishes the function Cwc24 in the splicing reaction (32). Here, we further analyzed the functional role of the ZF domain in splicing. We show that the ZF-motif is required for stable association of Cwc24 with the spliceosome. The C144A mutation affected the interaction of Cwc24 with the pre-mRNA, leading to aberrant U5–5′SS and U6–5′SS interactions. The interaction of Prp8 with the 5′ splice site was also less specific in the absence of Cwc24 or with Cwc24 carrying the C144A mutation. We also show that U5 and U6 are properly positioned at the 5′ splice site during Prp2-mediated spliceosome remodeling only when Cwc24 binds on the 5′ splice site, which prevents Prp8 from interacting with the 5′ splice site, suggesting an essential role for the Cwc24 ZF-domain in modulating specific interactions of U5, U6 and Prp8 with the 5′ splice site.

## MATERIALS AND METHODS

### Yeast strains

The following yeast strains were used: BJ2168 (MAT***a*** prc1 prb1 pep4 leu2 trp1 ura3), YSCC038 (MAT***a*** prc1 prb1 pep4 leu2 trp1 GAL1-CWC24::URA3) and EGY48 (MAT**a** trp1 ura3 his3 LEU::pLexop6-LEU2).

### Oligonucleotides

The following oligonucleotides were used for primer extension, affinity selection or sequencing:

Pre-II: TCTTACAGTTAAATGGGATGG

Pre-III: CAATTGGGACCGTGCAATTCT

Pre-V: GAGAAATCTCTCGAGCAATTG

U5-C_bio:_ bio-ACCCGGATGGTTCTGG

U6-A_bio_: bio-TCTCTTTGTAAAACGG

### Antibodies and reagents

Anti-Ntc20, anti-Prp8, anti-Prp2, anti-Spp2 and anti-Cwc24 are polyclonal antibodies produced by immunizing rabbits with corresponding recombinant proteins expressed in *Escherichia coli*. Full-length His-tagged Ntc20, full-length GST-tagged Spp2, full-length Cwc24, His-tagged Prp8 N-terminal fragment of amino acid residues 1–115 and Prp2 N-terminal fragment of amino acid residues 1–203 were used as antigens. Cwc24 and Prp2 N-terminal fragment were expressed as His-SUMO-tagged proteins with His-SUMO fragments removed after purification from Ni-NTA columns. Anti-V5 monoclonal antibody was purchased from Serotec Inc., and Protein A-Sepharose (PAS) was obtained from GE Healthcare Inc. Proteinase K was purchased from MD Bio Inc., and SuperScript III was purchased from Invitrogen.

### Splicing extracts, substrates and reactions

Splicing extracts were prepared according to Cheng *et al.* (35). Extracts metabolically depleted of Cwc24 were prepared according to Wu *et al.* (32). A truncated form of actin pre-mRNA, Ac/Cla, in which only five nucleotides were retained after the branch point (36), was used for probing U5-pre-mRNA and U6-pre-mRNA interactions by UV-crosslinking. Splicing substrates were synthesized *in vitro* with SP6 RNA polymerase using plasmid DNA pSPAct6-88 linearized with *Eco*RI or *Cla*I as templates. Splicing assays were carried out according to methods described previously by Cheng and Abelson (37). For UV-crosslinking of Cwc24 to pre-mRNA, pre-mRNA was labeled with ^32^P at ∼10^8^ dpm/pmole transcript.

### Immunodepletion, immunoprecipitation and spliceosome precipitation by streptavidin-Sepharose

Immunodepletion was performed by incubating 100 μl of splicing extracts with anti-Ntc20 antiserum coupled to 50 μl of protein A-Sepharose. We used 50 μl of anti-Ntc20 antiserum for depletion of NTC, and 150 μl of anti-Cwc24, 100 μl of anti-Cwc25, 200 μl of anti-Prp2 and 200 μl of anti-Spp2 antisera for depletion of Cwc24, Cwc25, Prp2 and Spp2, respectively. Immunoprecipitation was performed as previously described by Tarn *et al.* (38). The amounts of antibody used for precipitation of the spliceosome from 20 μl of splicing reaction were 1 μl for anti-Ntc20, 1.5 μl for anti-Prp8 antisera and 1 μg of purified anti-V5 antibody. The precipitates were then washed four times with 1 ml NET-2 buffer (50 mM Tris-HCl, pH 7.4, 150 mM NaCl and 0.05% NP-40). To isolate of Cwc24-V5- or Prp8-crosslinked products, each 1-ml of UV-irradiated splicing reaction mixture was precipitated with 50 μg of anti-V5 antibody or 150 μl of anti-Prp8 antiserum, respectively, conjugated to 500 μl of PAS following denaturation and neutralization.

### Purification of recombinant Cwc24

Purification of Cwc24 recombinant proteins was performed as described previously by Wu *et al.* (32).

### RNA–RNA crosslinking

UV-crosslinking of U5 and U6 to pre-mRNA was performed according to Chan *et al.* (20,21). Each 4 ml of the splicing reaction mixture was precipitated with anti-Ntc20 antibody (0.3 ml of serum coupled to 1 ml protein A-Sepharose). Bound materials were resuspended in 8 ml of Buffer E (12 mM HEPES-KOH, pH 7.9, 30 mM KCl, 3 mM MgCl_2_, 0.12 mM EDTA, and 12% (v/v) glycerol) and distributed onto a 10-cm culture dish. The dish was placed on ice under a UV_254 nm_ lamp at a distance of ∼10 cm in a UV Stratalinker 1800 (Stratagene Inc.), and irradiated at an energy level of 0.125 J/cm^2^. After deproteinization, RNA was precipitated and resuspended in 78 μl of H_2_O and then mixed with 10 μl of *E. coli* tRNA (10 mg/ml), 10 μl of Buffer S (0.2 M HEPES-KOH, pH 7.9, and 0.5 M KCl), 1 μl of RNasin (40 U/μl) and 1 μl of 5′-biotinylated oligonucleotide U5-C_bio_ or U6-A_bio_ at 300 μM. The mixture was boiled for 2 min, slow cooled to room temperature and precipitated with a 50 μl bed volume of streptavidin-Sepharose by incubation at 4°C for 1 h. The precipitate was heated for 2 min to release the selected RNAs, which were fractionated on a 5% polyacrylamide (29:1), 8 M urea gel (∼23 cm long, 20 cm wide and 0.4 mm thick). Individual crosslinked products were excised from gels and eluted. Following precipitation, the amount of RNA was estimated by counting in a Scintillation counter (LS6500; Beckman Coulter). For primer extension, 0.055–0.15 fmole of crosslinked RNA, 2 × 10^5^ cpm or 50 fmole of 5′-^32^P-labeled oligonucleotide Pre-III and 10 nmole each of four dNTPs was mixed in a 13 μl annealing mixture, heated to 65°C for 5 min, and incubated on ice for at least 1 min, and then mixed with a 7 μl prewarmed reaction mixture to give a final concentration of 50 mM Tris-HCl, pH 8.3, 75 mM KCl, 3 mM MgCl_2_, 5 mM dithiothreitol (DTT), 40 U of RNasin and 200 U of SuperScript III reverse transcriptase. The mixture was then incubated at 50°C for 1 h. Extension products were analyzed by electrophoresis on 10% polyacrylamide (19:1)-8 M urea gels (Thermo Scientific Owl S3S Aluminum-Backed Sequencer).

### RNA–protein crosslinking

For RNA–protein crosslinking, each 25 μl of the splicing reaction mixture with 2 nM pre-mRNA was spread onto a 10-cm culture dish placed on ice, irradiated with UV_254 nm_ at 0.8 J/cm^2^ (Stratalinker 1800), and then collected into a microcentrifuge tube. The sample was then added to a denaturation mix to give a final concentration of 1% SDS, 1% Triton X-100 and 0.1 M dithiothreitol (DTT). The mixture was boiled for 2 min and allowed to cool to room temperature in a water bath. The mixture was diluted 10-fold with a buffer containing 50 mM Tris-HCl pH 7.5, 300 mM NaCl, 1 mM EDTA, 0.05% NP-40 and 0.2 mg/ml tRNA, and subjected to immunoprecipitation. The precipitates were washed with the same buffer without tRNA and then digested with proteinase K, followed by phenol–chloroform extraction and ethanol precipitation. For primer extension analysis, RNA was isolated from 0.4 to 1 ml of UV-irradiated splicing reaction mixtures. Each RNA sample was mixed with 2 × 10^5^ cpm or 50 fmole of 5′-^32^P-labeled oligo Pre-II or Pre-V, denatured by heating at 65°C for 5 min, and then quickly chilled on ice. Primer extension reactions were carried out with SuperScript III reverse transcriptase at 50°C for 1 h. Reaction products were analyzed on 10% polyacrylamide (19:1)-8 M urea gels (Thermo Scientific Owl S3S Aluminum-Backed Sequencer).

### Pull-down assays

Recombinant V5-tagged Cwc24 mutant proteins bound to 10 μl of protein A-Sepharose beads conjugated with 3.5 μl anti-V5 antibody were incubated with *E. coli* lysates that expressed recombinant His-tagged SUMO-Brr2C for 1 h at 4°C. *Escherichia coli* lysates from 0.1 OD_600_ cells and 10 pmole of recombinant Cwc24 proteins were used for each reaction. Beads were washed four times with 1 ml of wash buffer (50 mM Tris-HCl, pH 7.4, 300 mM NaCl, and 0.05% NP-40). Washed beads were resuspended in 1 × SDS-PAGE loading buffer and boiled for 5 min prior to fractionation on SDS-PAGE.

### Yeast two-hybrid assays

To map Brr2 domains interacting with Cwc24, we fused Brr2 deletion mutants to the LexA-DNA binding domain in plasmid pEG202, and Cwc24 was fused to the Gal4-activation domain in plasmid pACT2. To map Cwc24 domains interacting with Brr2C, we fused Brr2C to the LexA-DNA binding domain in plasmid pEG202, and Cwc24 deletion mutants were fused to the Gal4-activation domain in plasmid pACT2. Each pair of plasmids was transformed into yeast strain EGY48 together with the reporter plasmid pSH18-34, and expression of β-galactosidase was assessed to determine protein–protein interactions.

## RESULTS

### The ZF domain is required for stable association of Cwc24 with the spliceosome

We previously reported that deletion of any region of Cwc24 that includes the ZF domain impairs splicing (32). Here, we examined whether splicing deficiency of the deletion mutants was due to an inability in binding the spliceosome. Splicing reactions were conducted in extracts prepared from cells metabolically depleted of Cwc24 (ΔCwc24) supplemented with recombinant V5-tagged Cwc24 proteins hosting various deletions at concentrations from 0.35 to 350 nM. Extracts were depleted of Spp2 prior to the reaction to prevent Prp2-mediated spliceosome remodeling (Figure 2A). Cwc24-associated spliceosomes were then precipitated with anti-V5 antibody (Figure 2B, lanes 6–9). As a control, the reaction mixture without Cwc24 supplement was precipitated with anti-Ntc20 antibody to isolate post-activation spliceosomes (lane 4). Our results showed that the abilities of Cwc24 mutants to bind the spliceosome were correlated with their splicing complementation activities of ΔCwc24 extracts (32). Full-length Cwc24 and the N1 mutant bound pre-catalytic spliceosomes to saturation levels at 3.5 nM, whereas the C1 mutant bound them with a lower affinity. Mutants lacking the ZF domain exhibited limited spliceosome binding (ΔZF and N2) or showed no binding at concentrations up to 350 nM (C2), and the C144A mutant had a similar affinity for spliceosomes as ΔZF. These results indicate that stable association of Cwc24 with the spliceosome requires the ZF domain with a functional zinc-binding site, and this association is further supported by the amino-terminal (N-terminal) sequence of Cwc24.

Cwc24 was previously shown to interact with the Ski2-like RNA helicase Brr2 (23,39). The carboxyl terminus (C-terminus) of Brr2 has been proposed to regulate splicing by interacting with several splicing factors, including Prp2, Prp16, Slu7 and Ntr2 (40,41). It is possible that deletion of or mutation of the ZF domain may affect the interaction of Cwc24 with Brr2 to prevent stable association of Cwc24 with the spliceosome. We first examined by two-hybrid assays if Cwc24 also interacts with the C-terminal domain of Brr2 (Brr2C), and we found that the C-terminal domain alone was sufficient for interaction with Cwc24 (Figure 2C). We then examined whether deletion of the ZF domain affected the interaction of Cwc24 with Brr2C. Results from both two-hybrid (Figure 2D) and pull-down assays using recombinant SUMO-fused Brr2C and V5-tagged Cwc24 mutants (Figure 2E) showed that the N-terminal region of Cwc24 (amino acid residues 1–137, N2) was sufficient for its interaction with Brr2C, and the ZF domain was not required for this interaction. This outcome indicates that the Cwc24–Brr2 interaction is not the primary factor responsible for stable association of Cwc24 with the spliceosome. Instead, it is possible that the interaction of Cwc24 with pre-mRNA is required for stable association of Cwc24 with the spliceosome.

### The ZF domain is important for mediating 5′ splice site selection

**Figure 3.**
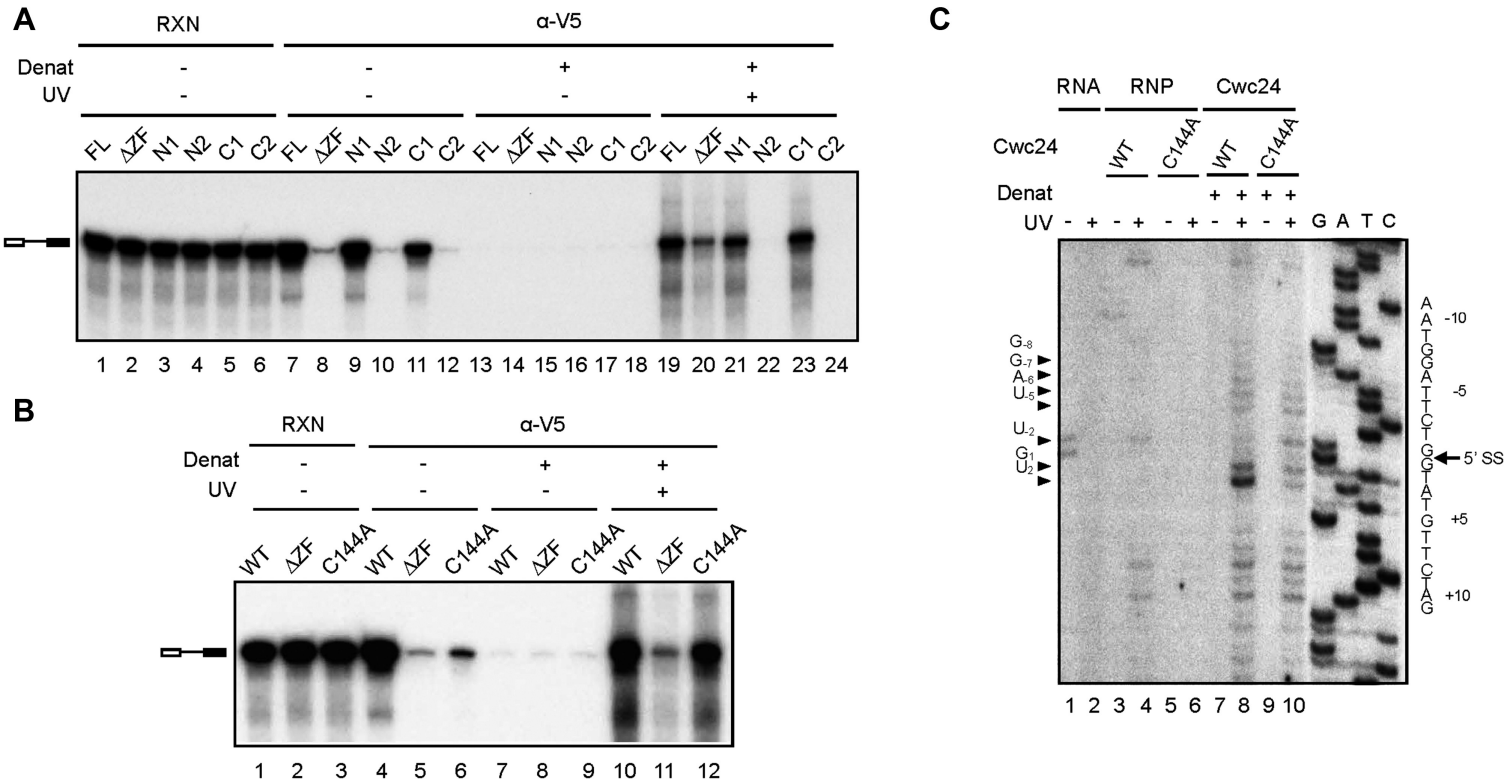
The ZF domain is required for specific interaction of Cwc24 with the 5′ splice site. (**A** and **B**) UV-crosslinking of Cwc24 deletion and C144A mutant proteins to pre-mRNA on the activated spliceosome. Splicing was carried out in Spp2-depleted ΔCwc24 extracts supplemented with recombinant V5-tagged Cwc24 proteins at a final concentration of 35 nM. The reaction mixtures were UV irradiated and then precipitated with anti-V5 antibody with prior denaturation. Reaction mixtures without UV irradiation were also precipitated with or without prior denaturation as controls. The relative amounts of samples loaded onto each lane are 1:10:1000 for RXN:no denat/IP:denat/IP. RXN, reaction mixture; Denat, denaturation. (**C**) Primer extension analysis of Cwc24 crosslinked sites. Splicing reactions and UV-crosslinking were carried out as in panel (B). RNA was extracted from the reaction mixtures (RNA), or after precipitation with anti-V5 antibody without (RNP) or with prior denaturant treatment (Cwc24) for primer extension using oligo Pre-V as a primer.

We previously demonstrated that Cwc24 directly interacts with the 5′ splice site of pre-mRNA on the activated spliceosome, contacting both the 5′ exon and intron sequences (32). A cryo-EM structure of the catalytically activated spliceosome also revealed close contacts between residues in the ZF domain and the 5′ splice site (22). We addressed whether deletion or mutation of the ZF domain would affect direct interaction of Cwc24 with the pre-mRNA by UV-crosslinking. Splicing was carried out in Spp2-depleted ΔCwc24 extracts supplemented with recombinant V5-tagged Cwc24 full-length or deletion mutant proteins at a final concentration of 35 nM, and the reaction mixtures were irradiated with UV_254 nm_. Following denaturation, Cwc24-pre-mRNA crosslinking was analyzed by immunoprecipitation with anti-V5 antibody (Figure 3A). Without crosslinking, the mutants lacking the ZF domain, i.e. ΔZF, N2 and C2, were barely associated with the spliceosome (Figure 3A; lanes 8, 10 and 12), mirroring the results shown in Figure 2B. N2 or C2 also did not crosslink to the pre-mRNA (lanes 22 and 24), suggesting that neither interacted with the pre-mRNA. In contrast, the ΔZF mutant did crosslink to the pre-mRNA, albeit at a substantially reduced level (lane 20). These results suggest that although the ZF domain is important for the interaction of Cwc24 with the pre-mRNA, other regions of the protein can also interact with the pre-mRNA, but with lower affinity. Furthermore, stable association of Cwc24 deletion mutants with the spliceosome was not linearly correlated with their abilities to interact with the pre-mRNA; a scenario further supported by the observation that the C144A mutant could crosslink to the pre-mRNA nearly as strongly as wild-type protein (Figure 3B; lane 12), but only weakly interacted with the spliceosome without crosslinking (lane 6). This outcome raises the possibility that the ZF mutant proteins might not interact with the pre-mRNA at the correct position to ensure their stable association with the spliceosome.

To establish whether weak association of C144A with the spliceosome was due to mis-localization of the mutant protein on the pre-mRNA, we mapped Cwc24-crosslinked sites by primer extension using a primer downstream of the 5′ splice site. In Figure 3C, we show that while wild-type Cwc24 crosslinked to the intron sequence primarily at positions +1 and +2 (lane 8), crosslinking of C144A mutant protein to the +2 position was greatly reduced (lane 10). Instead, the C144A mutant protein exhibited stronger crosslinking at the -2 position of the 5′ exon (lane 10), indicating that this mutant protein interacts with the 5′ splice site with reduced specificity. These results suggest that specific interaction of Cwc24 with the pre-mRNA for stable association with the pre-catalytic spliceosome requires a functional Cwc24 ZF domain.

### Stabilization of U5-5′SS and U6-5′SS interactions requires a functional ZF domain

**Figure 4.**
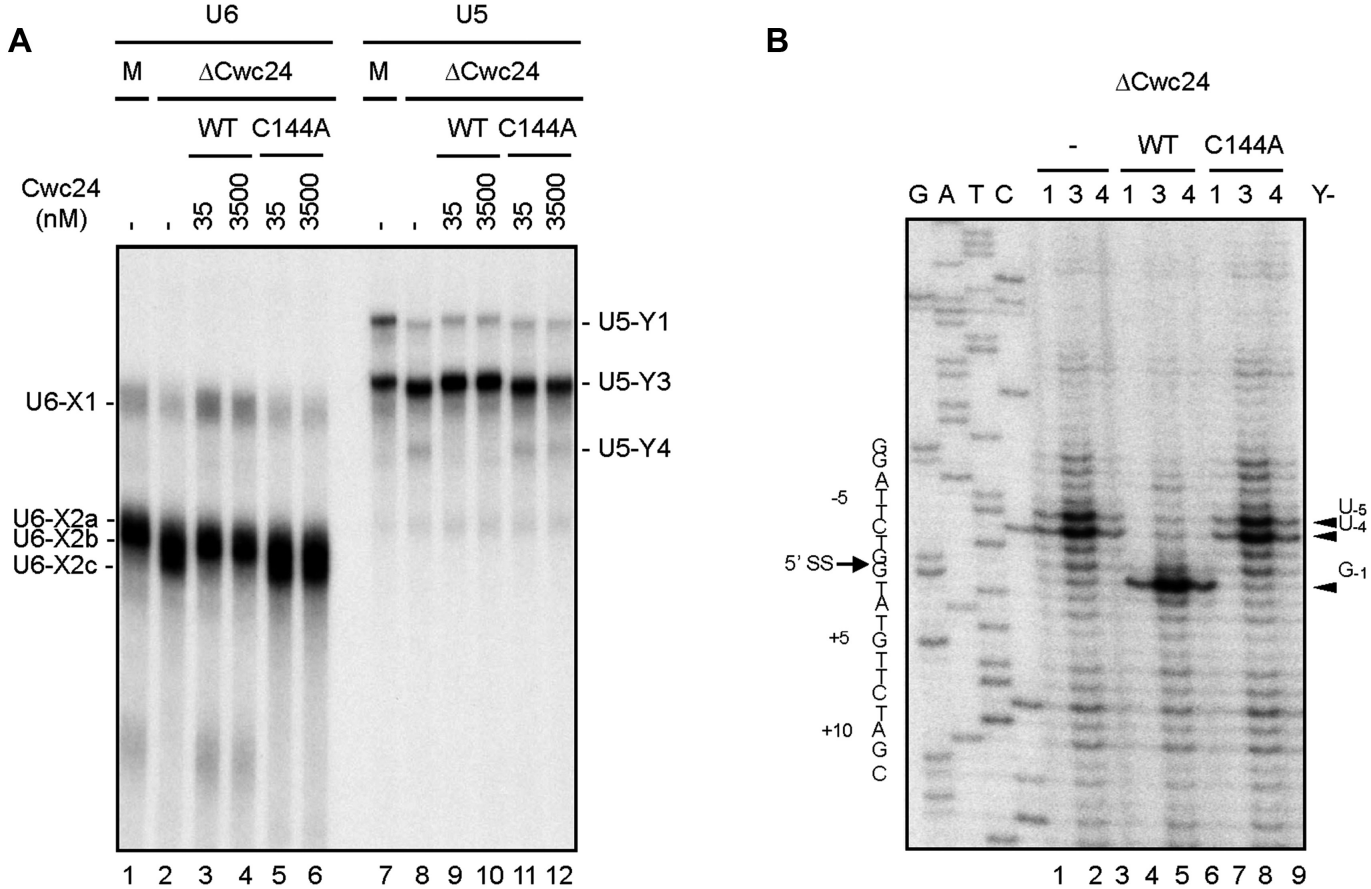
The Cwc24 C144A mutation altered U5-5′SS interactions. (**A**) Splicing was carried out in wild-type or ΔCwc24 extracts supplemented with or without recombinant V5-tagged wild-type or C144A Cwc24 protein at a concentration of 35 or 3,500 nM, and the reaction mixtures were precipitated with anti-Ntc20 antibody. Following UV-irradiation and deproteinization, U6- and U5-crosslinked products were affinity-selected with biotinylated oligos U6-A_bio_ and U5-C_bio_, respectively, and analyzed by PAGE. (**B**) Individual U5-crosslinked products from lanes 8, 9 and 11 of (A) were excised from the gel and RNA extracted for primer extension analysis using oligo Pre-III as a primer. 1, 3, 4 indicate Y1, Y3 and Y4, respectively.

We have previously shown that, in the absence of Cwc24, U5 and U6 fail to interact with the 5′ splice site at correct positions (32). To determine the role of the Cwc24 ZF domain in regulating the interactions of U5 and U6 with the pre-mRNA, we used the C144A mutant to analyze crosslinking of U5 and U6 with the pre-mRNA. Splicing was performed with Ac/Cla pre-mRNA (see ‘Materials and Methods’ section) in ΔCwc24 extracts supplemented with recombinant Cwc24 wild-type or C144A mutant proteins at concentrations of 35 nM or 3.5 μM. After irradiation with UV_254 nm_, U5/pre-mRNA and U6/pre-mRNA crosslinked products were selected with 5′-biotinylated oligonucleotides complementary to U5 and U6 snRNAs, respectively, as described previously (21). Figure 4A shows that unlike mock-treated (lanes 1 and 7) or wild-type Cwc24-supplemented (lanes 3, 4, 9 and 10) reactions, crosslinking patterns for C144A-supplemented reactions (lanes 5 and 6, 11 and 12) were similar to those of reactions carried out in the absence of Cwc24 (lanes 2 and 8), indicating that the C144A mutant is not functional in terms of promoting specific U5- and U6-pre-mRNA interactions. Crosslinking between U6 and the pre-mRNA in the absence of Cwc24 could be distinguished from that in the presence of wild-type Cwc24 by weaker X1 and X2a crosslinks with an extra product, X2c, which represents crosslinks between the conserved A_51_ residue of U6 with U_2_ of the 5′ splice site and with G_-1_ of the 5′ exon due to exposure of the 5′ splice site region when Cwc24 was absent (32). It is not surprising that we observed X2c in the reaction using the C144A mutant protein as this latter interacts less strongly and less specifically with the 5′ splice site (Figure 3C). In our U5-pre-mRNA crosslinking experiment (Figure 4A), crosslinked products Y1 and Y3 migrated slightly faster in reactions with either the C144A mutant or in the absence of wild-type Cwc24. Furthermore, an additional product, Y4, was also present in this experiment. Primer extension analysis revealed that crosslinked sites on the pre-mRNA were identical for reactions performed in the absence of Cwc24 and in the presence of the C144A mutant, shifting to the -4 and -5 positions of the 5′ exon (Figure 4B, lanes 1–3 and 7–9) rather than to the last nucleotide of the 5′ exon (lanes 4–6). These results suggest that the ZF domain is important for the function of Cwc24 in specifying the interactions of U5 and U6 with the 5′ splice site.

### The ZF domain is important for high-affinity binding of Cwc24 to the pre-mRNA and for the protein’s function after binding to the spliceosome

**Figure 5.**
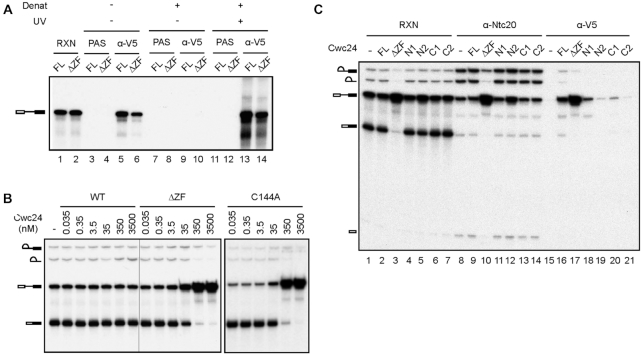
Cwc24 ZF mutants exhibit dominant-negative properties. (**A**) UV-crosslinking of Cwc24. Splicing was carried out in ΔCwc24 extracts, and the reaction mixtures were depleted of ATP before addition of recombinant V5-tagged wild-type or ΔZF Cwc24 protein at a final concentration of 3.5 μM. The reaction mixtures were UV irradiated and then precipitated with anti-V5 antibody with prior denaturation. Reaction mixtures without UV irradiation were also precipitated with or without prior denaturation as controls. The relative amounts of samples loaded onto each lane were 1:10:1000 for RXN:no denat/IP:denat/IP. RXN, reaction mixture; PAS, protein A-Sepharose; Denat, denaturation; FL, full-length. (**B**) Splicing reactions were carried out in the presence of recombinant Cwc24 wild-type, ΔZF or C144A mutant protein at concentrations ranging from 0.035 to 3500 nM. (**C**) Splicing was carried out in the presence of recombinant V5-tagged wild-type or mutant Cwc24 proteins at a concentration of 3.5 μM, and the reaction mixtures were precipitated with anti-Ntc20 or anti-V5 antibody; RXN, reaction.

Cwc24 ZF mutant proteins (ΔZF and C144A) were found to associate with the spliceosome when levels were increased to micromolar concentrations (Figure 2B), but they still do not promote the splicing reaction (32). At 3.5 μM, even the ΔZF mutant could crosslink to the pre-mRNA at efficiency only slightly lower than wild-type protein (Figure 5A; lanes 13 and 14), indicating that the ZF domain is not absolutely required for the interaction of Cwc24 with pre-mRNA. We further found that both the ΔZF and C144A mutants could interfere with the function of wild-type protein when they were separately added to splicing extracts at concentrations greater than 35 nM, displaying dominant-negative phenotypes (Figure 5B). None of the other deletion mutants exhibited dominant-negative phenotypes even at a concentration of 3.5 μM (Figure 5C; lanes 2–7), except for N2 that presented slight inhibition of the splicing reaction (lane 5) and resulted in an increase of unreacted spliceosomes following precipitation by anti-Ntc20 antibody (lane 12). Whereas the N2 mutant barely associated with the spliceosome (lane 19), the ΔZF mutant accumulated on the spliceosome in large amounts (lane 17) as revealed by precipitation with anti-V5 antibody. These results indicate that when present in excess amounts, the ZF mutants could compete with the endogenous wild-type protein to bind to the spliceosome, but they are not functional after binding to the spliceosome. Moreover, the C-terminal domain of Cwc24 protein stabilizes its interaction with the pre-mRNA. Together, our results suggest that the ZF domain is important for high-affinity binding of Cwc24 to the pre-mRNA and for its function after binding to the spliceosome.

### The ZF domain is important for 5′ splice site cleavage fidelity

**Figure 6.**
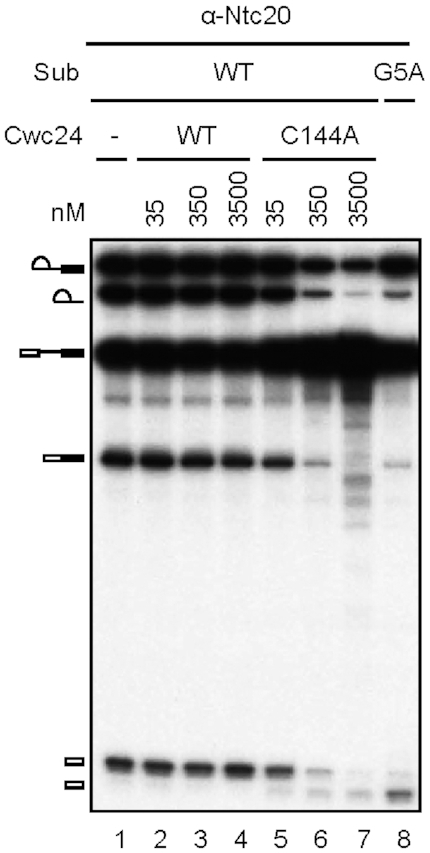
The dominant-negative Cwc24 ZF mutant exhibits aberrant cleavage at the 5′ splice site. Splicing was carried out with wild-type or G5A actin pre-mRNA in the absence or presence of 35 to 3500 nM wild-type or C144A V5-tagged Cwc24, and the reaction mixtures were precipitated with anti-Ntc20 antibody.

We have previously shown that depletion of Cwc24 by a genetic or antibody approach does not completely abrogate splicing activities in extracts, but does result in aberrant cleavage at the 5′ splice site (32). Since ZF mutant proteins do not properly interact with the 5′ splice site, it is possible that the ZF mutants would also elicit aberrant cleavage. To determine if the ZF domain is important for 5′ splice site cleavage fidelity, we performed splicing in the presence of C144A mutant protein at 35, 350 and 3500 nM, and enriched for activated spliceosomes by precipitation with anti-Ntc20 antibody. As shown in Figure 6, addition of increasing amounts of C144A mutant protein to the extract resulted in decreasing splicing activities but increased accumulation of activated spliceosomes, as well as increased aberrant cleavage of the 5′ splice site at the -5 position (lanes 5–7), as observed in splicing in Cwc24-depleted extracts (32) or with pre-mRNA carrying 5′ splice site G5A mutation (lane 8). These results suggest that the ZF mutant proteins might interfere with the binding of wild-type protein and inhibit cleavage at authentic 5′ splice site, and that the ZF domain is important for the fidelity of 5′ splice site selection.

### Cwc24 modulates the interaction of Prp8 with the 5′ splice site

**Figure 7.**
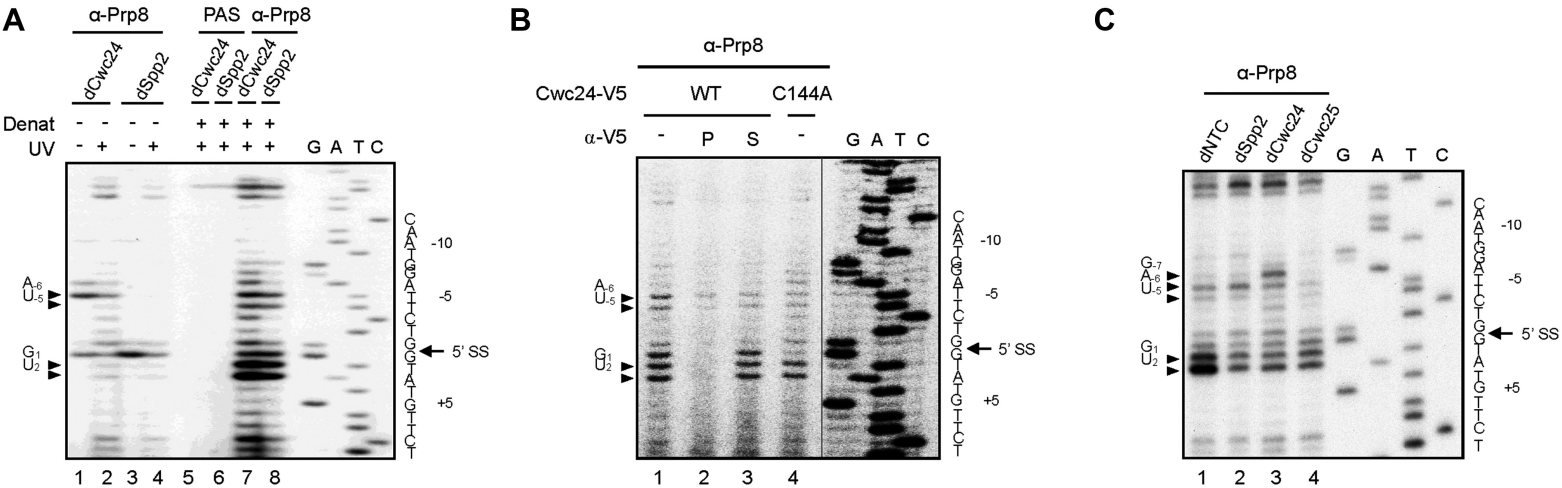
Cwc24 modulates the interaction of Prp8 with the 5′ splice site. Primer extension analyses to map Prp8-crosslinked sites at the 5′ splice site using oligo Pre-II as a primer. (**A**) Splicing was carried out in Cwc24-depleted or Spp2-depleted extracts. The reaction mixtures were UV-irradiated and precipitated with or without anti-Prp8 antibody-conjugated PAS with or without prior denaturant treatment. RNA was extracted for primer extension analysis; PAS, protein A-Sepharose. (**B**) Splicing was carried out in Prp2-depleted ΔCwc24 extracts supplemented with recombinant V5-tagged wild-type or C144A Cwc24 protein at a final concentration of 35 nM. The reaction mixtures were UV-irradiated (lanes 1 and 4), and Cwc24-associated (lane 2) and Cwc24-lacking (lane 3) spliceosomes were separated by precipitation with anti-V5 antibody. Following denaturant treatment, samples were precipitated with anti-Prp8 antibody and RNA was extracted for primer extension analysis; P, pellet; S, supernatant. (**C**) Splicing was carried out in NTC-depleted (lane 1), Spp2-depleted (lane 2), Cwc24-depleted (lane 3) or Cwc25-depleted (lane 4) extracts. The reaction mixtures were UV-irradiated, precipitated with anti-Prp8 antibody following denaturant treatment, and then RNA was extracted for primer extension analysis.

Prp8 has previously been shown to interact with the 5′ splice site before the branching reaction (18). We previously observed strong crosslinks of Prp8 at positions +1 and +2, as well as weaker crosslinks at -5 and -6 of the 5′ splice site, on post-activation spliceosomes formed on *ACT1* pre-mRNA (32). We also showed that Cwc24 crosslinks to positions +1, +2 and −2 of the 5′ splice site across the splice junction (32). In agreement with these results, the cryo-EM structures of activated spliceosomes reveal direct contacts of residues in the Cwc24 ZF domain with the +1 and +2 positions of the intron (22). It has also been shown by crosslinking analysis that the N-terminal region of Cwc24 interacts with the NTD1, RH and Jab1 domains of Prp8 on the B^act^ spliceosomes (23). Together, these results demonstrate close interactions of Cwc24, Prp8 and the 5′ splice site, raising the possibility that Cwc24 may regulate the interaction of Prp8 with the 5′ splice site on active spliceosomes. Consequently, we examined how Cwc24 affects crosslinking of Prp8 to the pre-mRNA. Splicing was performed in extracts depleted of Cwc24, or in Spp2-depleted extracts to accumulate Cwc24-containing post-activation spliceosomes. Following UV-irradiation and denaturation of the reaction mixtures, Prp8-crosslinked products were purified by immunoprecipitation with anti-Prp8 antibody and analyzed by primer extension for crosslinked sites (Figure 7A). Consistent with previous reports, we observed strong crosslinks of Prp8 at positions +1 and +2 of the intron sequence, and also at positions -5 and -6 on the 5′ exon. Nevertheless, Prp8 crosslinking was more specific in the presence of Cwc24 (lane 8), and spread through positions -3 to -9 in the absence of Cwc24 (lane 7) (tracing of lanes 7 and 8 shown in Supplementary Figure S2), suggesting that Cwc24 may promote specific interactions of Prp8 with the 5′ splice site.

Interestingly, our previous study demonstrated that Prp8 and Cwc24 displayed major crosslinks at positions +1 and +2 of the 5′ splice site on post-activation spliceosomes (32). Conceivably, Prp8 and Cwc24 may interact dynamically with the 5′ splice site so that either protein can contact these residues. Alternatively, Cwc24 may be present only on a fraction of the spliceosome, and its presence precludes the interaction of Prp8 with the 5′ splice site. To determine which of these possibilities are true, we analyzed Prp8 crosslinking on the spliceosomes selected for Cwc24 association (Figure 7B). To retain Cwc24 on the spliceosome, we performed splicing in Prp2-depleted ΔCwc24 extracts supplemented with V5-tagged wild-type Cwc24 protein at 35 nM. Following UV-irradiation, the Cwc24-associated spliceosomes were isolated by precipitation with anti-V5 antibody, and Prp8-crosslinked products were isolated by precipitation with anti-Prp8 antibody following denaturation of the spliceosome (lane 2). For comparison, total spliceosomes not selected for Cwc24 association (lane 1) or Cwc24-lacking spliceosomes, as in the supernatant fraction of the precipitates (lane 3), were also analyzed. Primer extension analyses revealed that crosslinking of Prp8 to the +1 or +2 position was not detected on the Cwc24-associated spliceosome, and crosslinking to positions -5 and -6 was also weakened (lane 2), indicating that the presence of Cwc24 hindered the Prp8-5′SS interaction. These findings also show that at least two populations of spliceosomes exist after spliceosome activation, i.e. those containing or lacking Cwc24, and the Prp8 crosslinks we observed on activated spliceosomes were from the population lacking Cwc24. We further examined how the Cwc24-C144A mutant affected the Prp8-5′SS interaction, and found that Prp8-5′SS crosslinking was more broadly distributed (Figure 7B; lane 4), as also observed in reactions performed in Cwc24-depleted extracts (Figure 7A; lane 7), suggesting that C144A mutant protein has no role in promoting specific interactions of Prp8 with the 5′ splice site.

We have previously shown that RNA base pairings in the RNA catalytic core are misarranged when spliceosomes are assembled in the absence of Cwc24 (32). The fact that the presence of Cwc24 on the activated spliceosome precludes Prp8 from interacting with the 5′ splice site suggests that Cwc24 might have to interact with the 5′ splice site before Prp8 during spliceosome activation in order to align U5 and U6 on the 5′ splice site region. On fully assembled spliceosome pre-B complex, the 5′ splice site is bound by U1 snRNP and has no contact with Prp8 (42–44). It is not known whether Prp8 interacts with the 5′ splice site upon the release of U1, since the 5′ portion of the pre-mRNA was not observed in the cryo-EM structures of yeast or human B-complex (42,45), which represents an intermediate of the spliceosome activation process in which U1 has been released but U4 is still retained. This scenario also suggests that the region of the 5′ splice site on the pre-mRNA might be in a dynamic state at this intermediate stage. To determine if Prp8 interacts with the 5′ splice site before Cwc24 is recruited to the spliceosome, we examined Prp8 crosslinking on spliceosomes formed in NTC-depleted extracts. NTC is required for spliceosome activation after the release of U4 to stabilize and specify interactions of U5 and U6 with the pre-mRNA (20,21). Cwc24 can only bind to the spliceosome in the presence of NTC (32). In Figure 7C, we show that Prp8 displayed a similar crosslinking pattern in NTC-depleted and Spp2-depleted extracts, but with pronounced crosslinks at the +1 and +2 positions (lanes 1 and 2), suggesting that Prp8 can interact with the 5′ splice site after U4 is released. This outcome also supports the view that the crosslinks at positions +1 and +2 we observed in Spp2-depleted extracts were generated from a subpopulation of spliceosomes prior to binding of Cwc24. Notably, only in Cwc24-depleted extracts were crosslinks more extensively dispersed (lane 3). We have previously shown that Prp2-mediated spliceosome remodeling can take place in the absence of Cwc24, but it results in aberrant interactions of U5 and U6 with the pre-mRNA (32). However, it is not clear whether Cwc24 regulates specific interactions of Prp8 with the 5′ splice site to direct positioning of U5 and U6 on the pre-mRNA during spliceosome remodeling, or those interactions are, more likely, a consequence of spliceosome remodeling upon dissociation of Cwc24. In either case, Prp8 may retain the same interaction pattern with the 5′ splice site after remodeling. Since Cwc25 is required for the first catalytic reaction after Prp2-mediated spliceosome remodeling and its depletion blocks the first reaction without affecting remodeling (46), we assessed crosslinking of Prp8 on post-Prp2 spliceosomes by performing splicing in Cwc25-depleted extracts. Indeed, crosslink signals were primarily associated with the +1 and +2 positions (lane 4), indicating that the interaction of Prp8 with the 5′ splice site is more specifically restricted to the +1 and +2 positions after spliceosome remodeling.

## DISCUSSION

Here, we show that the ZF motif of Cwc24 is required for stable association of Cwc24 with the spliceosome. When the ZF domain is deleted (as in the ΔZF or N2 mutants) or mutated (C144A), the association of Cwc24 with the spliceosome is greatly destabilized with only very low levels of spliceosomes being co-precipitated with Cwc24 even when the mutant proteins are present at concentrations more than 100-fold greater than for wild-type protein. Our crosslinking analyses revealed that the Cwc24 ZF mutants were capable of interacting with the pre-mRNA. At a concentration of 35 nM, the C144A mutant could crosslink to the pre-mRNA with an efficiency only slightly less than that of wild-type protein, albeit with lower specificity. In agreement with this result, electrophoretic mobility shift assays (EMSA) revealed that Cwc24 binds RNA nonspecifically, and the C144A mutation did not significantly alter its RNA binding capability (data not shown). Whereas wild-type Cwc24 crosslinked primarily to G1 and U2 of the 5′ splice site, crosslinking of the C144A mutant protein was relatively weak at these positions and was extended further upstream to the -2 position on the 5′ exon, indicating that an intact ZF structure is important for specific interaction of Cwc24 with the 5′ splice site. Cryo-EM structures of both human and yeast B^act^ complex reveal close positioning of several Cwc24 ZF residues to G1 and U2 of the 5′ splice site that may establish specific interactions between Cwc24 and the 5′ splice site (22,47). Nevertheless, deletion of the entire ZF domain resulted in only weak crosslinking, suggesting that the ZF domain might be the primary region interacting with pre-mRNA and that sequences other than the conserved residues contribute to the interaction. Members of the CCHC subclass of ZF have been reported to be involved in protein-protein interactions (48,49). Cwc24 has been shown to interact with several spliceosomal components on the B^act^ complex, including Prp2, Prp8 and Brr2 by crosslinking analysis (23). It is possible that Cwc24 may interact with these components to enable its positioning at the 5′ splice site. The C144A mutation may more profoundly affect the interaction of Cwc24 with adjacent protein components than it does interaction with the pre-mRNA, destabilizing the association of Cwc24 with the spliceosome so its interaction with the pre-mRNA is more dynamic and less specific.

Our Cwc24 ZF mutants showed a dominant-negative property in splicing reactions when provided at concentrations of 350 nM or more. These increasing protein concentrations resulted in enhanced association of the mutant proteins with the spliceosome. Therefore, although the mutant proteins do not tightly bind to the spliceosome, they interfere with spliceosomal binding of wild-type Cwc24 protein. Furthermore, they fail to promote the catalytic reaction upon binding to the spliceosome, with the residual splicing activities also resulting in aberrant cleavage at the 5′ splice site. These results suggest that an intact ZF is important for both stable association of Cwc24 with the spliceosome, and for its subsequent function in orchestrating the catalytic center of the spliceosome for branch formation.

Our two-hybrid and pull-down assays show that Cwc24 interacts with the C-terminal domain of Brr2. Brr2C has been shown to interact with several spliceosomal components, particularly for proteins required for the catalytic and disassembly steps (including DExD/H-box proteins Prp2 and Prp16), and may serve as a platform for recruiting splicing factors to the spliceosome (26,40,50). We have previously shown that Prp2 can be recruited to the spliceosome independently of Cwc24, but it requires the presence of Cwc24 for productive activity (32). Prp2 was crosslinked to the pre-mRNA, but only when the sequence downstream of the branchpoint (intron 3′ tail or i3′T), was longer than 20 bases. When the pre-mRNA had a shorter i3′T, Prp2 could not crosslink to the pre-mRNA, but it remained stably associated with the spliceosome, possibly by interacting with Brr2C. However, replacing the i3′T RNA sequence with DNA sequence beyond 20 bases downstream of the branchpoint destabilized the association of Prp2 with the spliceosome. It has been proposed that Prp2 is recruited to the spliceosome by interacting with Brr2C, and then it translocates to the pre-mRNA where it docks on the i3′T to mediate spliceosome remodeling (26). When Prp2 cannot translocate to the pre-mRNA due to a short i3′T, Prp2–Brr2 interaction allows stable association of Prp2 with the spliceosome. However, when the RNA sequence is replaced with DNA sequence in the i3′T, translocation of Prp2 to the DNA region results in destabilization of Prp2 due to its poor interaction with DNA (26). Similarly, Cwc24 may also be recruited to the spliceosome by interacting with Brr2C after the spliceosome has been activated. Cwc24 may then translocate to the 5′ splice site, and it is then released together with SF3a/b upon Prp2-mediated spliceosome remodeling. Cwc24 may behave similarly to Prp2, as suggested by the fact that the ZF mutant proteins cannot stably associate with the spliceosome after translocation to the 5′ splice site due to poor interactions with other components or low specific interaction with the pre-mRNA. This suggests that, like Prp2, the interaction of Cwc24 with the pre-mRNA may induce a conformational change in Cwc24 to destabilize its interaction with Brr2 so that Cwc24 can translocate to the pre-mRNA. However, if Cwc24 is not properly docked on the 5′ splice site after translocation, it cannot stably associate with the spliceosome.

Our deletion analyses revealed that Cwc24 interacts with Brr2C through its N-terminal region and independently of the ZF domain. Deletion of amino acid residues (aa) 1-127 of Cwc24, as in the C1 mutant, completely abolished its ability to interact with Brr2C, whereas the N-terminal segment of aa 1-137 is sufficient for the interaction. Although showing no growth defect, the C1 mutant is partially impaired for its function in the splicing reaction, requiring a >10-fold protein concentration to acquire comparable splicing activity as wild-type (32). The affinity of the C1 mutant for the spliceosome (Figure 2B) and for the pre-mRNA (Figure 3A) is concomitantly reduced. This suggests that although Cwc24 and Brr2C stably interact as free proteins, their interaction in the context of the activated spliceosome may be only transitory. It is worth noting that aa 1-125 of Cwc24 was not observable on the cryo-EM structure of the yeast B^act^ spliceosome (22), indicating that this region of the protein is a dynamic state. Cwc24 may translocate to the pre-mRNA immediately upon interaction with Brr2C, and becomes stably associated with the spliceosome only after docking on the 5′ splice site. The C1 mutant may contain sufficient yet suboptimal sequences for transient interaction not detected by two-hybrid or pull-down assays.

Previously, we showed that U5 and U6 interaction with the 5′ splice site on activated spliceosomes was aberrant in the absence of Cwc24 (32). U5 was observed to crosslink to the -4 and -5 positions of the 5′ splice site, as opposed to the -1 position when Cwc24 was present. Crosslinking of the A_51_ residue of U6 to positions −1 and +2 of the 5′ splice site was also only seen in the absence of Cwc24 (32). These results suggest that the structure of the RNA catalytic core is altered when the spliceosomes is assembled in the absence of Cwc24. Here, we further found that Cwc24 also modulates the interaction of Prp8 with the 5′ splice site during Prp2-mediated spliceosome remodeling. In the absence of Cwc24, crosslinking of Prp8 dispersed to the 5′ exon, suggesting that Prp8-5′SS interactions became more dynamic. The presence of the C144A mutant also resulted in a similar Prp8 crosslinking distribution pattern, possibly attributable to dynamic interactions of C144A with the 5′ splice site. Interestingly, Prp8 was not detected to crosslink to the 5′ splice site on the Cwc24-associated spliceosomes, except for rather weak crosslinks at the -5 and -6 positions, indicating that the presence of Cwc24 prevents Prp8 from contacting the 5′ splice site on activated spliceosomes. It is possible that after U4 is released and accompanied by binding of NTC, Cwc24 is immediately recruited to the spliceosome and binds to the 5′ splice site, thereby allowing proper positioning of U5 and U6 on the 5′ splice site. After remodeling of the spliceosome, Cwc24 is released, and its interactions with the +1 and +2 positions of the 5′ splice site are replaced by those of Prp8. The absence of NTC or Cwc24 allows Prp8 to interact immediately with the 5′ splice site upon the release of U4, but results in mislocalization of U5 and U6 on the 5′ splice site.

**Figure 8.**
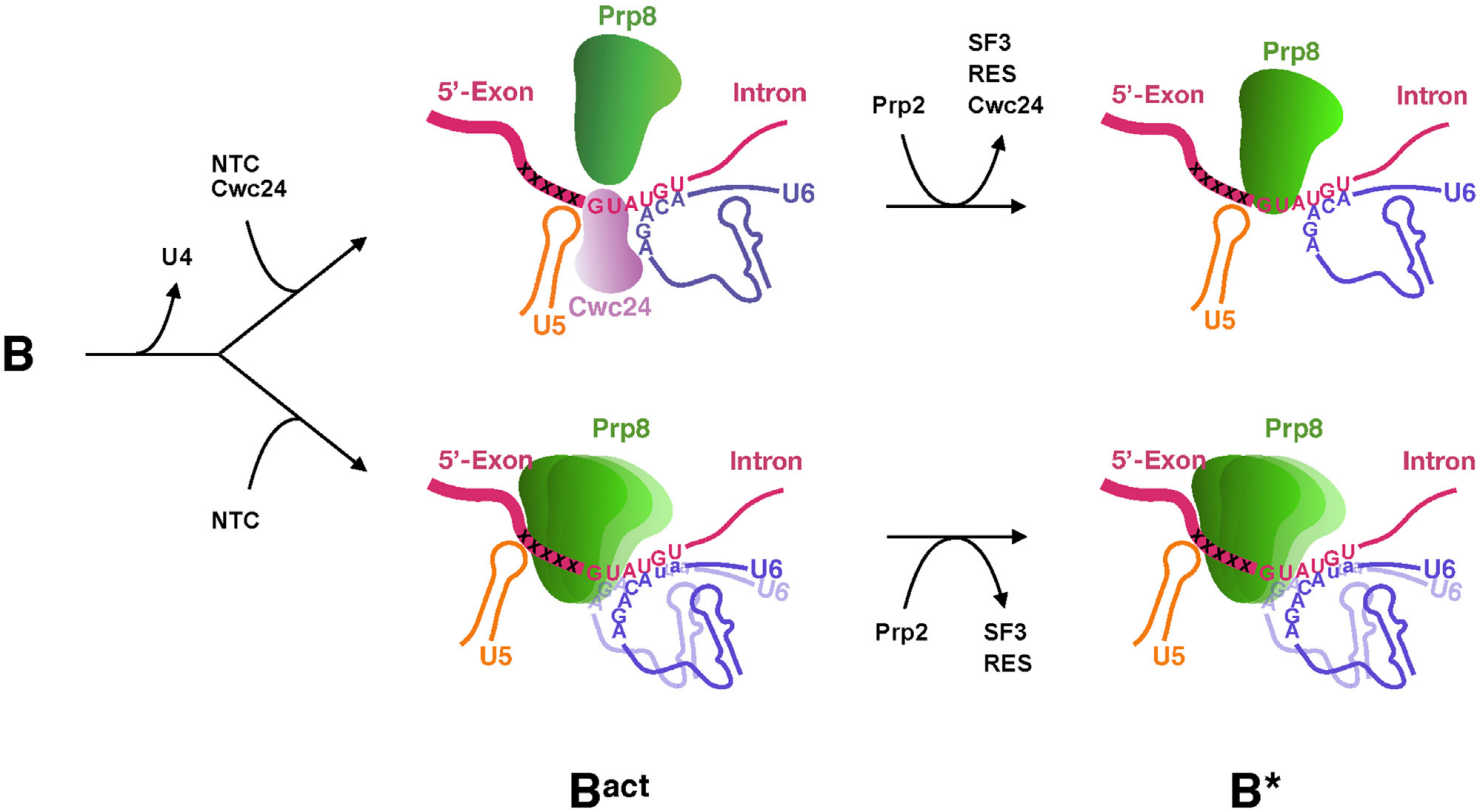
A model for how Cwc24 may modulate formation of the RNA catalytic core. During spliceosome activation, NTC is recruited to the spliceosome upon the release of U4. In the presence of Cwc24, which may bind to the spliceosome immediately upon binding of NTC (32), U5 and U6 interact with the 5′ splice site at correct positions to form an active catalytic core, with U5 interacting with the -1 position and U6 forming base pairs between residues ACAGA and UGU of the intron. The ZF domain of Cwc24 sequesters Prp8 from interacting with the GU residues of the 5′ splice site. The mode of RNA interactions is retained after Prp2-mediated spliceosome remodeling, which results in the release of Cwc24 to allow Prp8 to interact with the +1 and +2 positions of the 5′ splice site. When Cwc24 is absent during spliceosome activation, Prp8 interacts with the 5′ splice site in a dynamic manner over a wider region and U5-5′SS and U6-5′SS interactions are misarranged.

A model for how Cwc24 may mediate formation of the RNA catalytic core through Prp2-mediated spliceosome remodeling is presented in Figure 8. Upon the release of U4, Cwc24 may bind onto the 5′ splice site immediately upon the binding of NTC, with its ZF-domain contacting the +1 and +2 positions of the intron. A cryo-EM structure of the B^act^ complex reveals that the ZF-domain is positioned between the 5′ splice junction and the α-finger of Prp8, thus preventing Prp8 from contacting the 5′ splice site (22) (Supplementary Figure S1). Binding of Cwc24 to the 5′ splice site then positions U5 and U6 upstream and downstream, respectively, of that site to align RNA interactions at the catalytic core. After Prp2-mediated spliceosome remodeling, Cwc24 is displaced and Prp8 can then interact with the +1 and +2 positions of the 5′ splice site. At that stage, i.e. before step one factors Yju2 and Cwc25 enter the active site, the structure of the spliceosome is in a dynamic state, as revealed by resolutions of two different conformations of the cryo-EM structure for the B* complex (51). Moreover, the α-finger of Prp8 also was not observable in either of those structures. Subsequent binding of Yju2 and Cwc25 presumably stabilizes the structure of the active site and the interaction of the branchpoint with the 5′ splice site to promote the catalytic reaction. When spliceosome activation occurs in the absence of Cwc24 or when Cwc24 is not properly positioned at the 5′ splice site, Prp8 may interact with the 5′ splice site more dynamically over a wider range of sequences, pushing U5 to interact with the 5′ exon at the -4 and -5 positions. U6-5′SS interaction is also altered with the AUA sequence upstream of the ACAGA motif base paired with UGU of the intron 5′ splice site. This results in an inactive structure of the RNA catalytic core, which is retained after spliceosome remodeling.

The *Drosophila* Cwc24 ortholog *mdlc* has been shown to be required for the maintenance of neuronal differentiation. Knockdown of *mdlc* gene results in reduced levels and aberrant splicing of the *pros* transcript, indicating that Mdlc not only has a function in promoting splicing efficiency, but is also involved in splice-specific regulation (30). In light of our results that Cwc24 is required for proper alignment of RNA interactions at the catalytic core, how Mdlc mediates splicing regulation remains an interesting question. It is possible that although Cwc24 is strictly required for the splicing reaction in *Saccharomyces cerevisiae*, it may be more loosely required in other organisms where splicing regulation is ubiquitous. The level of the protein may impact splicing of various introns to different extents in fly and human, resulting in differential splicing patterns.

## Supplementary Material

gkz733_Supplemental_File
