# Supplementary material for: Functional analysis of Cwc24 ZF-domain in 5′ splice site selection
Source: Nucleic Acids Res. 2019 Aug 28;47(19):10327–39. doi: 10.1093/nar/gkz733 (PMC6821175; doi:10.1093/nar/gkz733)
Supplement: gkz733_Supplemental_File [file gkz733_supplemental_file.pdf]

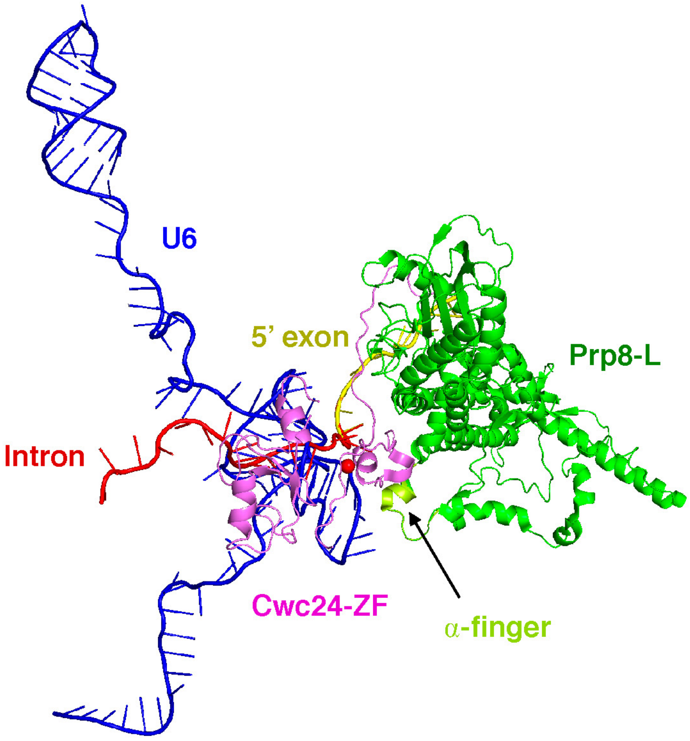

Figure S1. A cryo-EM structure model of Cwc24-5'SS-Prp8 interaction. Green, Prp8 Large domain; bright green, α-finger; pink, Cwc24 ZF domain; blue, U6; red, 5'SS region of intron; yellow, 5'ss region of 5' exon.

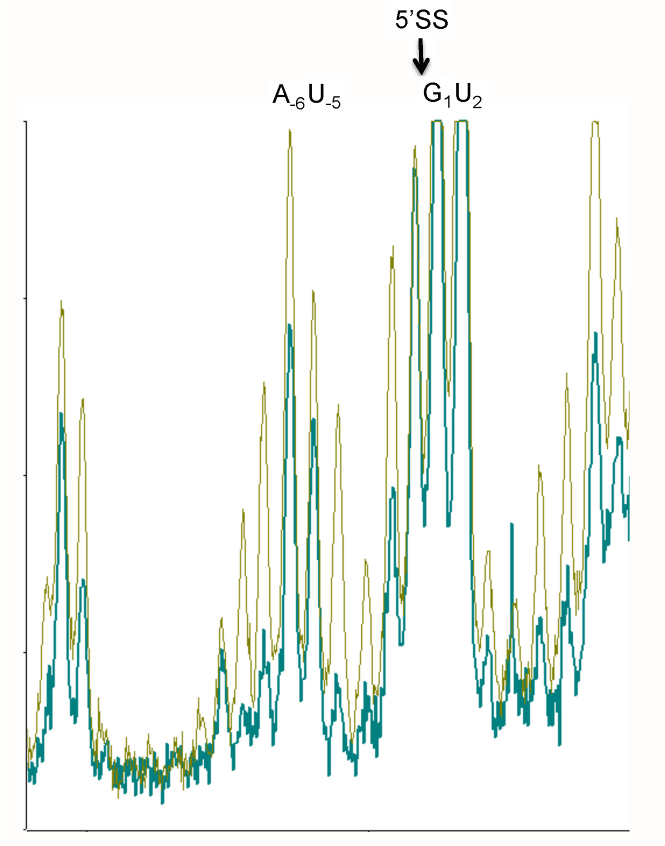

Figure S2. Tracing of primer extension for mapping of Prp8 crosslinking sites. Profile of scanning of autoradiogram from Figure 7A of splicing reactions performed in Cwc24-depleted (light green) and Spp2-depleted (green) extracts.
